# Evaluation of mild cognitive impairment genetic susceptibility risks in a Chinese population

**DOI:** 10.1186/s12888-022-03756-y

**Published:** 2022-02-08

**Authors:** Yelei Zhang, Xiaoyue Li, Yu Hu, Hongwei Yuan, Xiaodong Wu, Yating Yang, Tongtong Zhao, Ke Hu, Zhiqiang Wang, Guoqiang Wang, Kai Zhang, Huanzhong Liu

**Affiliations:** 1 Department of Psychiatry, Chaohu Hospital, Anhui Medical University, 64 North Chaohu Road, Hefei, 238000 China; 2 Anhui Psychiatric Center, Anhui Medical University, Hefei, 238000 China; 3 The Affiliated Kangning Hospital of Wenzhou Medical University, Wenzhou, Zhejiang, 325007 China; 4 Department of Psychiatry, Wuxi Mental Health Center, Nanjing Medical University, Wuxi, 214151 China

**Keywords:** Propensity score matching (PSM), Alzheimer disease (AD), Mild cognitive impairment (MCI), Polymorphisms

## Abstract

**Background:**

Mild cognitive impairment (MCI) is a kind of non-functional cognitive decline between normal aging and dementia. With the increase of individual age, the quality of cognitive function has become a more and more important topic. The study of gene loci in patients with MCI is essential for the prevention of dementia. In this study, we evaluate the gene polymorphism in Chinese Han patients with MCI by propensity score matching (PSM) and comparing them to healthy control (HC) subjects.

**Methods:**

Four hundred seventeen patients with mild cognitive impairment and 508 healthy people were included. The two groups were matched by applying one-to-one PSM, and the matching tolerance was set to 0.002. The matching covariates included gender,age,occupation,marital status,living mode. Then, a case-control associated analysis was conducted to analyze the genotype and allele frequencies of single nucleotide polymorphisms (SNPs) in the MCI group and the control group.

**Results:**

Three hundred eleven cases were successfully matched in each group, and there was no statistical difference on all the matching variables, gender, age, occupation, marital status, living mode between two groups after the match (*P* > 0.05). The allele frequency of bridging integrator 1(*BIN1*) rs7561528 showed minimal association with MCI in the Han Chinese population (*P* = 0.01). Compared with the healthy control (HC) group, A allele frequency of MCI group patients was significantly decreased. The genotype frequency of *BIN1* rs6733839 showed minimal association with MCI in the recessive model (*P* = 0.03). The genotype frequency of rs7561528 showed minimal association with MCI in the codominant, dominant, overdominant, and log-additive model (*P* < 0.05). The genotype frequencies of StAR-related lipid transfer domain 6 (*STARD6*) rs10164112 showed nominal association with MCI in the codominant, dominant, and log-additive model (P < 0.05). Unfortunately, the significant differences did not survive Benjamini-Hochberg false discovery rate correction (adjusted *P* > 0.05). The patients with *SPI1* rs1057233 may be the protective factor of MCI (OR = 0.733, 95%CI 0.625–0.859, *P* < 0.001), and patients with *APOE* rs10164112 may be a risk factor for MCI (OR = 1.323, 95%CI 1.023–1.711, *P* = 0.033).

**Conclusions:**

The polymorphisms of rs7561528, rs6733839 loci in the *BIN1* gene, and rs1057233 loci in the *SPI1* gene may be associated with the MCI in Chinese Han population. APOE gene was the risk factor of MCI, but further verification in a large sample population is still needed.

**Supplementary Information:**

The online version contains supplementary material available at 10.1186/s12888-022-03756-y.

## Introduction

Mild cognitive impairment (MCI) is a cognitive impairment mode between normal aging and dementia, which is characteristics of cognitive or some mild memory impairment [1]. In general, MCI patients can perform activities of daily living (ADLs) [2]. MCI could be a risk factor for Alzheimer’s disease (AD), Parkinson’s disease, dementia with Lewy bodies, and vascular cognitive impairment. The incidence of AD in MCI is 10 times higher than that in normal subjects. The clinical incidence of MCI ranges from 6 to 85%. On average, 10% of patients turn to dementia every year, and the incidence of dementia rises to 80 to 90% after 6 years [3]. MCI includes three subtypes: amnestic MCI (a-MCI), single-domain non-memory MCI (sd-MCI), and multiple-domain slightly impaired MCI (md-MCI) [4]. MCI is now considered to be a multifactorial disease, with the type of occupation, blood glucose levels, and hypertension all being associated with the development of MCI [5]. Among them, genetics is the most important influencing factor of MCI and also AD. Therefore, it is important to investigate the pathogenesis and etiology of MCI for the early diagnosis and treatment of AD.

Some genetic changes may contribute to dementia in some older people. Bridging Integrator Protein-1 (*BIN1*) gene, located on chromosome 2, is a member of the BAR family and participates in physiological processes such as cell endocytosis and actin activation [6]. In recent years, with the progress of molecular biology, Harold et al. proposed that the *BIN1* gene may be the pathogenic gene of AD. Some studies confirmed that the polymorphism of different *BIN1* loci genes is related to the pathogenesis of AD [7–9].

SPI1 (Recombinant Human Spi-1 Proto-Oncogene) encodes PU.1, a transcription factor that occupied a major position in myeloid cells’ development and function. The heritability of AD was abundant in the PU.1 cistrome, suggesting the presence of a myeloid PU.1 target gene network in AD. Huang et al. [10] that experimentally altered PU.1 levels were related to various biological processes such as the expression of multiple genes in myeloid cells and mouse microglial cells’ phagocytic activity. Besides, it was previously reported that the delayed onset of AD and the decreased expression of SPI1 in monocytes and macrophages were associated with the minor allele of rs1057233 (G). The above results suggest that the downregulation of SPI1 expression may reduce AD’s risk by adjusting gene expression and function in myeloid cells.

The Apolipoprotein E (APOE) is the main one of the apolipoproteins in plasma and is also involved in the nervous system’s growth and repair. Several studies have confirmed that the *APOE* gene is related to the incidence of AD and MCI, but the abnormal risk accounts for only 20%, suggesting that other genes are involved in the pathogenesis of AD [11, 12]. Furthermore, the combination of steroidogenic acute regulatory-related lipid transfer domain 6 (STARD6) rs10164112-T allele and *APOE* ε 4 allele raised the risk of developing AD [13]. However, the frequency of the rs10164112-T allele was significantly lower in the Korean population with AD than in the healthy population [14]. At present, there is no study to evaluate the status of rs10164112 in the Chinese Han population with MCI, while the relationship between single nucleotide polymorphisms (SNPs) and MCI is not clear.

Therefore, to better understand the relationship between these genes, we conducted a study that examined the distribution of SNPsof *BIN1*, *STARD6*, *RIN3*, *APOE*, *PICALM*, *SPI1*, *BZRAP1-AS*, *PFDN1/HBEGF*, *TMP21*, *MTHFR*, *TMEM106B*, *MC1R*, *CENPO*, *PVRL2* and *KL* genes to reveal the correlation between the polymorphisms and AD risk.

## Materials and methods

### Subjects

We performed a multicentric and prospective study. This study included 417 MCI patients and 508 healthy controls (HC) who were recruited between 2018 and 2020, who were all Han nationality over 50 years old in Wuxi City, Jiangsu Province, and its surrounding counties and communities. According to the revised Peterson criteria, MCI patients were diagnosed [15]. The subjects in the HC group must in keeping with the following conditions: illiteracy > 17 in the MMSE score scale, primary school > 20 points, junior high school, and above > 22, the CDR = 0 [16]. Each case meets the above diagnostic criteria. The clinical diagnosis was verified by a senior associate professor of psychiatry who had experience in using Structured Clinical Interview for DSM-IV.

The exclusion criteria were as follows: 1) People under the age of 50; 2) those with symptoms of psychosis or congenital mental retardation, history of head injury, severe endocrine diseases, severe cardiopulmonary, severe infectious diseases; 3) those who abuse alcohol or other substances. After a detailed description to the subjects or their representatives, written informed consents were obtained. In addition, the data on general demographics, such as age, sex, lifestyle, marital status, and occupation, were also surveyed.

### SNPs selection

Firstly, we selected SNPs in the public HapMap database (ftp://ftp.ncbi.nlm.nih.gov/hapmap/). The criterion for selecting SNPs is that the minor allele frequency (MAF) ≥0.05 and r2 ≥ 0.8 in Beijing’s Han Chinese population (HCB). We selected eighteen SNPs for genotyping were screened out for analysis, specific information for each gene is given in Table 5 in Supplementary Material. Genes such as *RIN3* and were selected due to their strong association with AD [17, 18]. We expect to find a link between these genes and MCI in Han Chinese populations as well.

### Genotyping

All participants fasted for at least 8 h prior to blood collection. Each participant collected about 5 ml of peripheral blood in ethylenediamine tetraacetic acid (EDTA). Tiangen DNA isolation kit was used to extract genomic DNA from venous blood. The selected SNPs were genotyped by TaqMan SNP Genotyping Assay and ABI PRISM 7900 sequence detection system equipped with SDS2.1 software. In order to quality monitoring, a blind method was used to perform genotype analysis on participants’ status. Ten percent of the samples were genotyped once more, showed a coincidence rate of 99.2%. Two independent researchers scored the genotype data doubly. Deviation from the expectation of Hardy-Weinberg equilibrium (HWE) was evaluated in this queue.

### Propensity score matching (PSM)

Rubin and Rosenbaum first proposed PSM in 1983 to eliminate the impact of confounding factors on the retrospective study results [18]. Propensity score matching used R (version 3.6.0) for 1: 1 matching and graph processing, and the matching tolerance is 0.002. Matching variables include gender, age, occupation, marriage, living mode.

### Statistical analysis

We assessed HWE, the genotype frequency measurements and the allele frequency with SHEsis software (http://analysis.bio-x.cn/myAnalysis.php). We apply the Benjamini-Hochberg false discovery rate correction to account for multiple tests. SNPStats (https://www.snpstats.net/start.htm) was mainly used to assess the connection between SNPs and MCI risk under five genetic models (including dominant, dominant, recessive, dominant and logarithmic additive models). Then, a Logistic regression analysis was performed with SPSS 24.0.

## Results

### General demographic and characteristics after propensity score matching

A total of 925 eligible subjects were asked to participate. We included 311 pairs of data by propensity score matching analysis. A total of 311 healthy controls (HC) were enrolled, including 168 females. The average age was 66.05 ± 6.21 years (Table 1). MCI patients mean age was 66.08 ± 6.91 years, includes 163 females and 148 males. Statistically significant differences with regard to age and occupation before PSM between patients and controls. Therefore, we adjusted these differences with propensity score matching. Results of propensity score-matched analyses are displayed in Table 1. After PSM, there was no significant difference in the clinical characteristics of the two groups.

**Table 1:** Baseline comparison of influencing factors before and after PSM

	Before PSM				After PSM			
	MCI(*n* = 417)	HC(*n* = 508)	*P*	SMD	MCI(*n* = 311)	HC(*n* = 311)	*P*	SMD
Gender-Female (%)	237(56.8)	260(51.2)	0.09	0.11	163(52.4)	168(54.0)	0.75	0.03
Age	67.06 ± 7.28	65.76 ± 6.26	0.004*	0.19	66.08 ± 6.91	66.05 ± 6.21	0.95	0.005
Occupation(%)			< 0.001*	0.68			0.94	0.07
Clerk	19(4.6)	67(13.2)			19(6.1)	16(5.1)		
Craftsman	255(61.2)	308(60.6)			225(72.3)	233(74.9)		
Farmers or unemployed	107(25.7)	33(6.5)			33(10.6)	30(9.6)		
Technical personnel	29(7.0)	66(13.0)			27(8.7)	24(7.7)		
Other occupations	7(1.7)	34(6.7)			7(2.3)	8(2.6)		
Marriage - having a spouse (%)	368(88.2)	465(91.5)	0.12	0.11	281(90.4)	283(91.0)	0.89	0.02
Living mode (%)			0.74	0.05			0.92	0.03
Live alone	20(4.8)	23(4.5)			14(4.5)	14(4.5)		
Live with spouse	230(55.2)	293(57.7)			171(55.0)	176(56.6)		
Live with posterity	167(40.0)	192(37.8)			126(40.5)	121(38.9)		
*APOE* genotype			χ^2^	P			χ^2^	P
			5.63	0.34			5.84	0.32
ε2/2	4(1.0)	6(1.3)			4(1.4)	5(1.7)		
ε2/3	54(13.9)	54(11.6)			42(14.6)	36(12.4)		
ε2/4	5(1.3)	6(1.3)			2(0.7)	4(1.4)		
ε3/3	265(68.5)	321(68.7)			197(68.4)	193(66.6)		
ε3/4	56(14.5)	80(17.1)			40(13.9)	52(17.9)		
ε4/4	3(0.8)	0(0.0)			3(1.0)	0(0.0)		
*APOE* allele			0.62	0.73			0.65	0.72
ε2	67(8.7)	72(7.7)			52(9.0)	50(8.6)		
ε3	640(82.7)	776(83.1)			476(82.6)	474(81.7)		
ε4	67(8.7)	86(9.2)			48(8.3)	56(9.7)		

*P < 0.05

### Gene polymorphisms of APOE

No significant association was observed for the distribution of APOE gene subtypes ε2/2, ε2/3, ε2/4, ε3/3, ε3/4, ε4/4, and their alleles ε2, ε3, ε4 between the two groups (*P* > 0.05, Table 1).

### The distribution difference of genotype and allele frequencies between MCI group and HC group on different genes

Except for *RIN3* rs10498633, *MTHFR* rs1801133, *MC1R* rs2228479, *PVRL2* rs6859, and *APOE* rs7412, the remaining SNP genotypes of both groups were in HWE (Table 2). The analysis showed that rs7561528 alleles frequency in *BIN1* differs between MCI patients and controls (χ 2 = 6.39, *p* = 0.01). The A allele frequency was 58 (9.6%) in the MCI group and 86 (14.3%) in the healthy control (HC) group. The distribution frequencies of AA, AG, and GG genotypes at rs7561528 loci in the MCI group were 5 (1.7%), 48 (15.8%), and 250 (82.5%), respectively. In HC group, they were 7 (2.3%), 72 (23.9%) and 222 (73.8%), respectively. When we compared the three genotypes’ distribution frequency between these two groups, we find a significant difference (χ 2 = 6.79, *P* = 0.03). Moreover, the T allele frequency at rs10164112 polymorphism in *STARD6* was higher in MCI cases than in controls, and the difference was statistically significant (χ2 = 5.30, *P* = 0.02). However, no difference remains significant after Benjamini-Hochberg’s false discovery rate correction (adjusted *P* > 0.05). Comparing genotype and allele frequencies remaining of the SNPs across the overall sample of MCI patients and controls showed no significant differences. Specific information on each SNP is given in Table 5 in the Supplementary Material.

**Table 2:** Allele and genotype frequency of the SNPs

Closest gene	SNP	n	Allele n(%)		*χ*^*2*^	*P*	Minor Allele	Genotype n(%)			*χ*^*2*^	*P*	*P* of HWE^a^
*BIN1*	rs6733839		C	T	3.44	0.06	T	CC	CT	TT	4.66	0.10	
	MCI	293	342(58.4)	244(41.6)				97(33.1)	148(50.5)	48(16.4)			0.50
	HC	276	292(52.9)	260(47.1)				81(29.3)	130(47.1)	65(23.6)			0.36
*BIN1*	rs7561528		A	G	6.39	**0.01**^b^	A	AA	AG	GG	6.79	**0.03**^b^	
	MCI	303	58(9.6)	548(90.4)				5(1.7)	48(15.8)	250(82.5)			0.14
	HC	301	86(14.3)	516(85.7)				7(2.3)	72(23.9)	222(73.8)			0.69
*RIN3*	rs10498633		G	T	0.88	0.35	T	GG	GT	TT	4.64	0.10	
	MCI	309	543(87.9)	75(12.1)				238(77.0)	67(21.7)	4(1.3)			0.77
	HC	311	557(89.5)	65(10.5)				254(81.7)	49(15.8)	8(2.6)			0.01
*PICALM*	rs10792832		A	G	0.36	0.55	A	AA	AG	GG	0.93	0.63	
	MCI	307	248(40.4)	366(59.6)				45(14.7)	158(51.5)	104(33.9)			0.23
	HC	309	260(42.1)	358(57.9)				54(17.5)	152(49.2)	103(33.3)			0.87
*SPI1*	rs1057233		C	T	0.89	0.35	C	CC	TC	TT	0.92	0.63	
	MCI	251	155(30.9)	347(69.1)				25(10.0)	105(41.8)	121(48.2)			0.75
	HC	285	161(28.2)	409(71.8)				25(8.8)	111(38.9)	149(52.3)			0.51
*TMP21*	rs12435391		A	G	1.18	0.28	A	AA	AG	GG	1.77	0.41	
	MCI	308	113(18.3)	503(81.7)				12(3.9)	89(28.9)	207(67.2)			0.53
	HC	306	98(16.0)	514(84.0)				12(3.9)	74(24.2)	220(71.9)			0.08
*MTHFR*	rs1801133		C	T	0.46	0.50	T	CC	CT	TT	2.14	0.34	
	MCI	304	354(58.2)	254(41.8)				98(32.2)	158(52.0)	48(15.8)			0.23
	HC	311	374(60.1)	248(39.9)				100(32.2)	174(55.9)	37(11.9)			0.003
*TMEM106B*	rs1990622		C	T	0.001	0.98	T	CC	CT	TT	0.17	0.92	
	MCI	308	421(68.3)	195(31.7)				144(46.8)	133(43.2)	31(10.1)			0.97
	HC	309	422(68.3)	196(31.7)				142(46.0)	138(44.7)	29(9.4)			0.58
*MC1R*	rs2228479		A	G	0.46	0.50	A	AA	AG	GG	2.92	0.23	
	MCI	307	141(23.0)	473(77.0)				20(6.5)	101(32.9)	186(60.6)			< 0.001
	HC	309	132(21.4)	486(78.6)				14(4.5)	104(33.7)	191(61.8)			0.001
*CENPO*	rs6669072		C	T	0.03	0.86	T	CC	CT	TT	0.41	0.81	
	MCI	310	500(80.6)	120(19.4)				203(65.5)	94(30.3)	13(4.2)			0.61
	HC	309	496(80.3)	122(19.7)				198(64.1)	100(32.4)	11(3.6)			0.71
*PVRL2*	rs6859		A	G	0.01	0.92	A	AA	AG	GG	1.49	0.47	
	MCI	310	195(31.5)	425(68.5)				30(9.7)	135(43.5)	145(46.8)			0.86
	HC	309	196(31.7)	422(68.3)				24(7.8)	148(47.9)	137(44.3)			0.06
*STARD6*	rs10164112		C	T	5.30	**0.02**^b^	T	CC	CT	TT	5.79	0.06	
	MCI	307	427(69.5)	187(30.5)				144(46.9)	139(45.3)	24(7.8)			0.23
	HC	309	466(75.4)	152(24.6)				171(55.3)	124(40.1)	14(4.5)			0.15
*APOE*	rs7920721		A	G	0.72	0.40	G	AA	AG	GG	1.59	0.45	
	MCI	308	467(75.8)	149(24.2)				178(57.8)	111(36.0)	19(6.2)			0.76
	HC	310	457(73.7)	163(26.3)				165(53.2)	127(41.0)	18(5.8)			0.32
*APOE*	rs429358		C	T	0.82	0.37	C	CC	CT	TT	2.92	0.23	
	MCI	294	50(8.5)	538(91.5)				3(1.0)	44(15.0)	247(84.0)			0.51
	HC	294	59(10.0)	529(90.0)				1(0.3)	57(19.4)	236(80.3)			0.21
*APOE*	rs7412		C	T	0.01	0.93	T	CC	CT	TT	1.24	0.54	
	MCI	300	545(90.8)	55(9.2)				251(83.7)	43(14.3)	6(2.0)			0.02
	HC	299	544(91.0)	54(9.0)				254(84.9)	36(12.0)	9(3.0)			< 0.001
*KL*	rs9536314		G	T	0.00	1.00	G	GT	TT		0.00	1.00	
	MCI	310	2(0.3)	618(99.7)				2(0.6)	308(99.4)				0.96
	HC	310	2(0.3)	618(99.7)				2(0.6)	308(99.4)				0.96
*BZRAP1-AS1*	rs2632516		C	G	0.74	0.39	C	CC	CG	GG	1.03	0.60	
	MCI	307	274(44.6)	340(55.4)				55(17.9)	164(53.4)	88(28.7)			0.16
	HC	308	290(47.1)	326(52.9)				65(21.1)	160(51.9)	83(26.9)			0.46
*PFDN1/* *HBEGF*	rs11168036		G	T	0.48	0.49	T	GG	GT	TT	0.51	0.77	
	MCI	307	348(56.7)	266(43.3)				98(31.9)	152(49.5)	57(18.6)			0.89
	HC	307	360(58.6)	254(41.4)				106(34.5)	148(48.2)	53(17.3)			0.91

^a^ *HWE* Hardy-Weinberg equilibrium test

^b^ After Benjamini-Hochberg false discovery rate correction, *P* > 0.05

Under five-inheritance models, the age and sex factors of MCI patients and controls were analyzed by unconditional Logistic regression. In the recessive model, there was a minimal association between the genotype frequency of *BIN1* gene rs6733839 and MCI. (CC-CT vs. TT). Similarly, the genotype frequency of *BIN1* rs7561528, the distribution frequencies of the codominant model (GG vs. AG), dominant model (GG vs. AG-AA), overdominant model (GG-AA vs. AG), and there was a nominally significant difference between the two groups with respect to log-additive model (*P* < 0.05, Table 3). As for the *APOE* rs10164112 polymorphism, the genotype frequencies showed a nominal association with MCI in the dominant model (CC vs. CT-TT), codominant model (CC vs. TT), log-additive model. However, when applying Benjamini-Hochberg to correct the false discovery rate, the association is not significant (*P* > 0.05 after correction).

**Table 3:** Logistic regression analysis of SNPs

SNP	Inheritance model		OR (95%CI)	*P*^f^
rs6733839	Codominant^a^	CC vs CT	0.96(0.65–1.40)	0.10
		CC vs TT	0.62(0.38–1.00)	
	Dominant^b^	CC vs CT-TT	0.84(0.59–1.20)	0.35
	Recessive^c^	CC-CT vs TT	0.64(0.42–0.96)	**0.03**^f^
	Overdominant^d^	CC-TT vs CT	1.15(0.83–1.60)	0.40
	Log-additive^e^		0.80(0.64–1.02)	0.07
rs10164112	Codominant	CC vs CT	1.33(0.96–1.85)	**0.05**^f^
		CC vs TT	2.04(1.02–4.10)	
	Dominant	CC vs CT-TT	1.41(1.02–1.93)	**0.04**^f^
	Recessive	CC-CT vs TT	1.79(0.91–3.54)	0.09
	Overdominant	CC-TT vs CT	1.24(0.90–1.70)	0.19
	Log-additive		1.38(1.06–1.79)	**0.02**^f^
rs10498633	Codominant	GG vs TG	1.46(0.97–2.20)	0.10
		GG vs TT	0.53(0.16–1.79)	
	Dominant	GG vs TG-TT	1.33(0.90–1.97)	0.15
	Recessive	GG-TG vs TT	0.50(0.15–1.66)	0.24
	Overdominant	GG-TT vs TG	1.48(0.98–2.23)	0.06
	Log-additive		1.17(0.83–1.65)	0.37
rs10792832	Codominant	GG vs AG	1.03(0.72–1.46)	0.63
		GG vs AA	0.83(0.51–1.34)	
	Dominant	GG vs AG-AA	0.98(0.70–1.37)	0.89
	Recessive	GG-AG vs AA	0.81(0.53–1.25)	0.35
	Overdominant	GG-AA vs AG	1.09(0.80–1.50)	0.57
	Log-additive		0.93(0.74–1.17)	0.55
rs11168036	Codominant	GG vs TG	1.11(0.78–1.58)	0.78
		GG vs TT	1.16(0.73–1.85)	
	Dominant	GG vs TG-TT	1.12(0.80–1.57)	0.50
	Recessive	GG-TG vs TT	1.09(0.72–1.65)	0.69
	Overdominant	GG-TT vs TG	1.05(0.77–1.45)	0.74
	Log-additive		1.08(0.86–1.36)	0.50
rs12435391	Codominant	GG vs AG	1.28(0.89–1.84)	0.41
		GG vs AA	1.08(0.47–2.45)	
	Dominant	GG vs AG-AA	1.25(0.89–1.77)	0.20
	Recessive	GG-AG vs AA	1.00(0.44–2.28)	0.99
	Overdominant	GG-AA vs AG	1.27(0.89–1.83)	0.19
	Log-additive		1.17(0.88–1.56)	0.29
rs1801133	Codominant	CC vs CT	0.93(0.65–1.32)	0.34
		CC vs TT	1.32(0.79–2.21)	
	Dominant	CC vs CT-TT	1.00(0.71–1.40)	0.98
	Recessive	CC-CT vs TT	1.39(0.87–2.20)	0.16
	Overdominant	CC-TT vs CT	0.85(0.62–1.17)	0.32
	Log-additive		1.09(0.86–1.39)	0.47
rs1990622	Codominant	CC vs CT	0.95(0.68–1.33)	0.92
		CC vs TT1.06	1.06(0.60–1.85)	
	Dominant	CC vs CT-TT	0.97(0.71–1.33)	0.85
	Recessive	CC-CT vs TT	1.08(0.63–1.85)	0.77
	Overdominant	CC-TT vs CT	0.94(0.69–1.30)	0.71
	Log-additive		1.00(0.78–1.27)	0.99
rs2228479	Codominant	GG vs AG	1.00(0.71–1.40)	0.55
		GG vs AA	1.48(0.72–3.02)	
	Dominant	GG vs AG-AA	1.05(0.76–1.46)	0.75
	Recessive	GG-AG vs AA	1.48(0.73–2.99)	0.27
	Overdominant	GG-AA vs AG	0.97(0.69–1.35)	0.84
	Log-additive		1.10(0.84–1.43)	0.50
rs2632516	Codominant	GG vs CG	0.97(0.67–1.40)	0.61
		GG vs CC	0.80(0.50–1.28)	
	Dominant	GG vs CG-CC	0.92(0.64–1.31)	0.64
	Recessive	GG-CG vs CC	0.82(0.55–1.22)	0.32
	Overdominant	GG-CC vs CG	1.06(0.77–1.46)	0.72
	Log-additive		0.90(0.71–1.14)	0.38
rs6669072	Codominant	CC vs CT	0.92(0.65–1.29)	0.80
		CC vs TT	1.17(0.51–2.68)	
	Dominant	CC vs CT-TT	0.94(0.68–1.31)	0.72
	Recessive	CC-CT vs TT	1.20(0.53–2.74)	0.66
	Overdominant	CC-TT vs CT	0.91(0.65–1.28)	0.58
	Log-additive		0.98(0.74–1.30)	0.88
rs6859	Codominant	GG vs AG	0.86(0.62–1.20)	0.48
		GG vs AA	1.19(0.66–2.13)	
	Dominant	GG vs AG-AA	0.91(0.66–1.25)	0.56
	Recessive	GG-AG vs AA	1.28(0.73–2.24)	0.39
	Overdominant	GG-AA vs AG	0.84(0.61–1.15)	0.28
	Log-additive		0.99(0.77–1.27)	0.93
rs7561528	Codominant	GG vs AG	0.59(0.39–0.88)	**0.03**^f^
		GG vs AA	0.65(0.20–2.08)	
	Dominant	GG vs AG-AA	0.59(0.40–0.88)	**0.01**^f^
	Recessive	GG-AG vs AA	0.71(0.22–2.29)	0.57
	Overdominant	GG-AA vs AG	0.59(0.39–0.89)	**0.01**^f^
	Log-additive		0.65(0.46–0.92)	**0.01**^f^
rs7920721	Codominant	AA vs AG	0.81(0.58–1.13)	0.44
		AA vs GG	0.98(0.50–1.94)	
	Dominant	AA vs AG-GG	0.83(0.60–1.14)	0.25
	Recessive	AA-AG vs GG	1.07(0.55–2.09)	0.84
	Overdominant	AA-GG vs AG	0.81(0.58–2.09)	0.20
	Log-additive		0.89(0.69–1.16)	0.39
rs9536314		TT vs TG	1.01(0.14–7.27)	0.99
rs1057233	Codominant	TT vs CT	1.17(0.81–1.67)	0.63
		TT vs CC	1.24(0.68–2.27)	
	Dominant	TT vs CT-CC	1.18(0.84–1.66)	0.34
	Recessive	TT-CT vs CC	1.16(0.65–2.07)	0.63
	Overdominant	TT-CC vs CT	1.13(0.80–1.59)	0.50
	Log-additive		1.13(0.87–1.47)	0.35
rs429358	Codominant	TT vs CT	0.74(0.48–1.14)	0.23
		TT vs CC	2.80(0.29–27.31)	
	Dominant	TT vs CT-CC	0.77(0.51–1.18)	0.24
	Recessive	TT-CT vs CC	2.96(0.30–28.90)	0.32
	Overdominant	TT-CC vs CT	0.73(0.47–1.13)	0.16
	Log-additive		0.83(0.55–1.24)	0.36
rs7412	Codominant	CC vs CT	1.21(0.75–1.95)	0.51
		CC vs TT	0.66(0.23–1.88)	
	Dominant	CC vs CT-TT	1.10(0.71–1.71)	0.67
	Recessive	CC-CT vs TT	0.64(0.22–1.83)	0.40
	Overdominant	CC-TT vs CT	1.23(0.76–1.98)	0.40
	Log-additive		1.01(0.71–1.45)	0.96

*CI* confidence interval, *OR *odds ratio

^a^ Codominant: major allele homozygotes vs. heterozygotes

^b^ Dominant: major allele homozygotes vs. heterozygotes + minor allele homozygotes

^c^ Recessive: major allele homozygotes + heterozygotes vs. minor allele homozygotes

^d^ Overdominant: major allele homozygotes + minor allele homozygotes vs. heterozygotes

^e^ Log-additive: major allele homozygotes vs. heterozygotes vs. minor allele homozygotes

^f^ After Benjamini-Hochberg false discovery rate correction, *P* > 0.05

### Forest map of the effect of gene polymorphism on MCI

The results of binary logistic regression are shown in Table 4 and Additional file 2: Fig. 1. Rs1057233 in *SPI1* was the protective factor for MCI (OR = 0.742, 95%CI 0.633–0.868, *P* < 0.001) and rs10164112 in *STARD6* was the risk factor for MCI (OR = 1.310, 95%CI 1.013–1.694, *P* = 0.040). In our study, the presence or absence of carrying *APOE*ε4 was not statistically associated with the occurrence of MCI (*P* > 0.05).

**Table 4:** Multiple logistic regression analysis of mild cognitive impairment

	B	S.E.	Wald	df	*P*	Exp(B)	95%C.I.for EXP(B)	
							Lower	Upper
*STARD6* rs10164112	0.270	0.131	4.235	1	0.040*	1.310	1.013	1.694
*SPI1* rs1057233	− 0.299	0.081	13.796	1	< 0.001*	0.742	0.633	0.868
*APOE*ε4	−0.192	0.228	0.709	1	0.400	0.826	0.529	1.290

*P < 0.05

## Discussion

The bridging integrator 1 (*BIN1*), also known as amphibian protein 2. It has been reported that its expression level in the brain tissue of patients with AD is increased [19] and is significantly related to the number of the pathology of neurofibrillary tangle (NFT) [20]. BIN1 is a major risk factor for late-onset AD (LOAD) [21]. BIN1 levels in patients with sporadic AD decreased by 87% compared with those in the non-dementia control group [22]. In addition, BIN1 protein plays a regulatory role in endocytosis, transport, immune system, calcium transient, and apoptosis [6]. BIN1 might be involved in the pathogenesis of AD in several ways, but the exact role is not clear. Since BIN1 affects the endocytosis pathway and intracellular transport mediated by Clathrin [23], it is speculated that it may be involved in the occurrence and development of AD through amyloid precursor proteins (APP) and APOE [6]. In addition, the interaction between BIN1 and tau protein was confirmed in both in vivo and in vitro models. It is speculated that BIN1 may be related to tau’s formation, the main pathological change of AD [8, 24].

There is evidence that BIN1 was related to episodic memory performance (in the context of genotyping patterns that involve binding to additional AD genes) [25]. Raj et al. was found that the expression level of BIN1 was affected by the *BIN1* rs7561528 locus [26]. This polymorphic genotype was also closely related to right hippocampal atrophy [27]. Harold et al. Large-scale GWAS analysis of Caucasian AD patients found three *BIN1* SNPs, including *BIN1* rs7561528, were significantly associated with AD [28]. This is also confirmed by another large-scale GWAS [29]. Significant association between LOAD and rs7561528 polymorphism in Han Chinese population [30]. Similar results were obtained by a Meta-analysis of the relationship between AD in East Asians and Caucasians. Rs7561528 A-allele carriers possibly as a protective factor of AD susceptibility in all genetic patterns in mixed populations and allele and dominance patterns in East Asian populations, and individuals with A/G heterozygote genotype in these two populations are not susceptible to AD [31]. Previous studies have confirmed the association between *APOE* ε4 carriers and rs7561528 [32]. Meanwhile, a meta-analysis of 74,046 participants found that *BIN1* rs6733839 SNP was related to AD [33]. Greenbaum et al. observed an association between well-established AD susceptibility SNP rs6733839 and episodic memory, and it can an important genetic risk factor for MCI among elderly individuals [34]. Based on the above studies, we have reason to believe that rs6733839 and rs7561528 gene polymorphism occupy a vital position in the pathogenesis of MCI by affecting the expression of BIN1. Our research found that two SNPs (rs6733839 and rs7561528) may be related to the pathogenesis of MCI among the elderly after using the one-to-one propensity score matching to reduce the hybrid effect. At present, the research on the role of BIN1 in AD is still in its infancy, which can understand the biological mechanism of cognitive decline and provide a new opportunity to find treatment sites. Additional functional genetic and independent replication analyses are necessary to elucidate these association epidemiological correlations.

After phagocytosis of amyloid-beta (Aβ), microglia initiate the activation of NALP3 inflammatory bodies and then activate caspase-1, which leads to the release of interleukin 1β (IL-1β) and promotes the occurrence of the inflammatory response [35]. NLRP3 inflammatory bodies are activated in AD, MCI brain, and APPPS1 mice. This activation may use substrates other than IL-1β to reduce Aβ phagocytosis and lead to Aβ deposition. Therefore, *NLRP3* and *Caspase-1* gene deletions can interfere with AD’s progression and improve cognitive function by blocking the formation of NLRP3/Caspase--1 inflammatory body [36]. The transcriptome and proteome analysis of microglia indicates that microglia homeostasis characteristics will be disturbed during aging and pathological state [37]. As a transcription factor, SPI1 directly regulates other AD-related genes expressed in myeloid cells such as microglia. *SPI1* may amplify the genetic variation of other AD-related myeloid genes and regulate neuroprotective or neurotoxic microglial phenotype equilibrium. Huang et al. found that *SPI1* rs1057233 and its labeled SNPs may regulate AD risk through changes in SPI1 expression and may represent potential disease sites [10]. Notably, rs1057233 was previously found to be associated with systemic lupus erythematosus [38], body mass index [39], and proinsulin levels [40], indicating that it may be involved in the link between AD, MCI, immune cell dysfunction, obesity, and diabetes.

New research suggests that neurosteroids such as diethylstilbestrol may be a new treatment for AD, indicating that lipid metabolism occupied a significant position in AD [41, 42]. Some studies have found that the behavior of STARD6 is similar to the steroidogenic acute regulatory protein (StAR), which controls the rate-limiting step of neurosteroid synthesis [35, 43].

Furthermore, STARD6 appeared in the hippocampus formation in rats, and its level was increased after pilocarpine-induced hippocampal neuron injury of rats [44]. The multivariate logistic regression model showed that *STARD6* rs10164112 was significantly related to AD in the Korean population [14]. Yin et al. found that the rs10164112-T allele combined with the *APOE*ε4 allele, resulting in an increased danger of AD [45]. Although the functional contribution of STARD6 in MCI is unknown, considering its role in AD, it may be involved in the pathogenesis of MCI. We found that the T allele of rs10164112 polymorphism was associated with a higher risk of MCI. The logistic regression model showed that the correlation was also significant in the total sample. Thus, it is possible to suggest that STARD6 participates in the pathogenesis of MCI.

At present, some researchers have reported that *APOE* ε4 may be a risk element for AD, and *APOE* ε2 may be a protective factor for AD [46–48]. However, our study showed that there was no difference in the subtypes of ε2/2, ε2/3, ε2/4, ε3/3, ε3/4, ε4/4, and alleles ε 2, ε 3, ε 4 of *APOE* gene between the two groups, which may be related to the fact that populations from different regions may have genetic heterogeneity of MCI.

## Conclusion

In summary, the present study demonstrated that *SPI1* and *BIN1* variation may be the potential targets for MCI treatment and supported that *STARD6* contributes to the risk of MCI. These results are helpful to understand the relationship among the pathogenesis, clinical diagnosis, and the SNPs of MCI in the Han population of southeastern China and provide directions for future research.

### Limitations

Several limitations of this study should be taken into consideration and discussed. Firstly, this is a cross-sectional study, so it didn’t consider the order of exposure and the timing of outcome and the causal relationship between exposure and outcome. Secondly, it is worth noting that age and occupation were significant differences between the MCI patients’ controls before PSM. Our study eliminated these variables to reflect the role of genes, but previous studies have confirmed the influence of age and gender on MCI. As a disease affected by many factors, other variables such as marriage, nutritional and mental status should be added when collecting clinical data of MCI. Some other genes, such as rs744373 in *BIN1*, were also found to be significantly associated with the occurrence of MCI in the Han Chinese population, but our study did not include all relevant SNPs [49]. Therefore, in the future, we need to conduct large-scale genetic studies in several populations to replicate the results and explore whether different variables together with genes affect morbidity. Finally, our diagnostic criterion for inclusion of MCI patients was Petersen criteria. Although this inclusion criterion is more in line with the clinical diagnosis, it does not allow for the identification of categories of MCI due to lack of sensitivity and inclusiveness [50]. In addition, we have not analyzed in depth the relationship between MCI subgroups and gene polymorphisms. Therefore, in the future, we will make further subdivision of MCI in combination with neuropsychological and brain scans.

## Supplementary Information

**Additional file 1: Supplementary Table 5.** Detailed information of SNPs in fourteen GWAS-linked genes^a^.

**Additional file 2: Fig. 1** OR (95%CI) forest map of the effect of gene polymorphism on MCI.

## Data Availability

The datasets generated during and/or analyzed during the current study are available from the corresponding author on request.

## Abbreviations

PSM: Propensity Score Matching
MCI: Mild cognitive impairment
AD: Alzheimer disease
SDAT: Senile dementia of Alzheimer type
SNPs: Single nucleotide polymorphisms
MMSE: Mini-mental State Examination
HIS: Hachinski Ischemic Scale
HDS: Hastgawa Dementia Scale
CDR: Clinical Dementia Rating
HWE: Hardy-Weinberg equilibrium test

## References

[1] Stephan BCM, Savva GM, Brayne C, John Bond BA, McKeith IG, Matthews FE (2010). Optimizing mild cognitive impairment for discriminating dementia risk in the general older population. Am J Geriatr Psychiatry.

[2] Puente AN, Terry DP, Faraco CC, Brown CL, Miller LS (2014). Functional impairment in mild cognitive impairment evidenced using performance-based measurement. J Geriatr Psychiatry Neurol.

[3] Eshkoor SA, Hamid TA, Mun CY, Ng CK (2015). Mild cognitive impairment and its management in older people. Clin Interv Aging.

[4] Edwards ER, Spira AP, Barnes DE, Yaffe K (2009). Neuropsychiatric symptoms in mild cognitive impairment: differences by subtype and progression to dementia. Int J Geriatr Psychiatry.

[5] Jia J, Zhou A, Wei C, Jia X, Wang F, Li F, Wu X, Mok V, Gauthier S, Tang M (2014). The prevalence of mild cognitive impairment and its etiological subtypes in elderly Chinese. Alzheimers Dement.

[6] Tan MS, Yu JT, Tan L (2013). Bridging integrator 1 (BIN1): form, function, and Alzheimer's disease. Trends Mol Med.

[7] Carrasquillo MM, Belbin O, Hunter TA, Ma L, Bisceglio GD, Zou F, Crook JE, Pankratz VS, Sando SB, Aasly JO (2011). Replication of BIN1 association with Alzheimer's disease and evaluation of genetic interactions. J Alzheimers Dis.

[8] Chapuis J, Hansmannel F, Gistelinck M, Mounier A, Van Cauwenberghe C, Kolen KV, Geller F, Sottejeau Y, Harold D, Dourlen P (2013). Increased expression of BIN1 mediates Alzheimer genetic risk by modulating tau pathology. Mol Psychiatry.

[9] Shen L, Thompson PM, Potkin SG, Bertram L, Farrer LA, Foroud TM, Green RC, Hu X, Huentelman MJ, Kim S (2014). Genetic analysis of quantitative phenotypes in AD and MCI: imaging, cognition and biomarkers. Brain Imaging Behav.

[10] Huang KL, Marcora E, Pimenova AA, Di Narzo AF, Kapoor M, Jin SC, Harari O, Bertelsen S, Fairfax BP, Czajkowski J (2017). A common haplotype lowers PU.1 expression in myeloid cells and delays onset of Alzheimer’s disease. Nat Neurosci.

[11] Yu JT, Tan L, Hardy J (2014). Apolipoprotein E in Alzheimer's disease: an update. Annu Rev Neurosci.

[12] Zhu XC, Wang HF, Jiang T, Lu H, Tan MS, Tan CC, Tan L, Tan L, Yu JT (2017). Alzheimer’s disease neuroimaging I: effect of CR1 genetic variants on cerebrospinal fluid and neuroimaging biomarkers in healthy, mild cognitive impairment and Alzheimer's disease cohorts. Mol Neurobiol.

[13] Desikan RS, Schork AJ, Wang Y, Thompson WK, Dehghan A, Ridker PM, Chasman DI, McEvoy LK, Holland D, Chen CH (2015). Polygenic overlap between C-reactive protein, plasma lipids, and Alzheimer disease. Circulation.

[14] Kim YJ, Paik JW, Kang WS, Kim SK, Lee KJ, Na HR, Park HJ, Kim JW, Lim HK, Park JK (2016). Genetic association of STARD6 polymorphisms with Alzheimer's disease in a Korean population. J Neurol Sci.

[15] Winblad B, Palmer K, Kivipelto MV, Jelic LF, Wahlund L-O, Nordberg A, Ckman LB, Albert M, Almkvist O (2004). Mild cognitive impairment--beyond controversies, towards a consensus: Report of the international working group on mild cognitive impairment. J Intern Med.

[16] Wang R, Yan Z, Liang Y, Tan EC, Cai C, Jiang H, Song A, Qiu C (2015). Prevalence and patterns of chronic disease pairs and multimorbidity among older Chinese adults living in a rural area. PLoS One.

[17] Stage E, Duran T, Risacher SL, Goukasian N, Do TM, West JD, Wilhalme H, Nho K, Phillips M, Elashoff D (2016). The effect of the top 20 Alzheimer disease risk genes on gray-matter density and FDG PET brain metabolism. Alzheimers Dement (Amst).

[18] Jun GR, Chung J, Mez J, Barber R, Beecham GW, Bennett DA, Buxbaum JD, Byrd GS, Carrasquillo MM, Crane PK (2017). Transethnic genome-wide scan identifies novel Alzheimer's disease loci. Alzheimers Dement.

[19] Karch CM, Jeng AT, Nowotny P, Cady J, Cruchaga C, Goate AM (2012). Expression of novel Alzheimer's disease risk genes in control and Alzheimer's disease brains. PLoS One.

[20] Holler CJ, Davis PR, Beckett TL, Platt TL, Webb RL, Head E, Murphy MP (2014). Bridging integrator 1 (BIN1) protein expression increases in the Alzheimer's disease brain and correlates with neurofibrillary tangle pathology. J Alzheimers Dis.

[21] Naj AC, Jun G, Beecham GW, Wang LS, Vardarajan BN, Buros J, Gallins PJ, Buxbaum JD, Jarvik GP, Crane PK (2011). Common variants at MS4A4/MS4A6E, CD2AP, CD33 and EPHA1 are associated with late-onset Alzheimer's disease. Nat Genet.

[22] Glennon EB, Whitehouse IJ, Miners JS, Kehoe PG, Love S, Kellett KA, Hooper NM (2013). BIN1 is decreased in sporadic but not familial Alzheimer's disease or in aging. PLoS One.

[23] Karch CM, Goate AM (2015). Alzheimer's disease risk genes and mechanisms of disease pathogenesis. Biol Psychiatry.

[24] Kingwell K (2013). Alzheimer disease: BIN1 variant increases risk of Alzheimer disease through tau. Nat Rev Neurol.

[25] Barral S, Bird T, Goate A, Farlow MR, Diaz-Arrastia R, Bennett DA, Graff-Radford N, Boeve BF, Sweet RA (2012). Genotype patterns at PICALM, CR1, BIN1, CLU, and APOE genes are associated with episodic memory. Neurology.

[26] Raj T, Shulman JM, Keenan BT, Chibnik LB, Evans DA, Bennett DA, Stranger BE, De Jager PL (2012). Alzheimer disease susceptibility loci: evidence for a protein network under natural selection. Am J Hum Genet.

[27] Wang HF, Wan Y, Hao XK, Cao L, Zhu XC, Jiang T, Tan MS, Tan L, Zhang DQ, Tan L (2016). Bridging integrator 1 (BIN1) genotypes mediate Alzheimer's disease risk by altering neuronal degeneration. J Alzheimers Dis.

[28] Harold D, Abraham R, Hollingworth P, Sims R, Gerrish A, Hamshere ML, Pahwa JS, Moskvina V, Dowzell K, Williams A (2009). Genome-wide association study identifies variants at CLU and PICALM associated with Alzheimer's disease. Nat Genet.

[29] Kamboh MI, Demirci FY, Wang X, Minster RL, Carrasquillo MM, Pankratz VS, Younkin SG, Saykin AJ (2012). Alzheimer’s Disease Neuroimaging I, Jun G et al: Genome-wide association study of Alzheimer's disease. Transl Psychiatry.

[30] Li HL, Yang P, Liu ZJ, Sun YM, Lu SJ, Tao QQ, Guo QH, Wu ZY (2015). Common variants at Bin1 are associated with sporadic Alzheimer's disease in the Han Chinese population. Psychiatr Genet.

[31] Zhou F, Haina D (2017). The bridging integrator 1 gene rs7561528 polymorphism contributes to Alzheimer's disease susceptibility in east Asian and Caucasian populations. Clin Chim Acta.

[32] Lee JH, Cheng R, Barral S, Reitz C, Medrano M, Lantigua R, Jimenez-Velazquez IZ, Rogaeva E, St George-Hyslop PH, Mayeux R (2011). Identification of novel loci for Alzheimer disease and replication of CLU, PICALM, and BIN1 in Caribbean Hispanic individuals. Arch Neurol.

[33] Lambert JC, Ibrahim-Verbaas CA, Harold D, Naj AC, Sims R, Bellenguez C, DeStafano AL, Bis JC, Beecham GW, Grenier-Boley B (2013). Meta-analysis of 74,046 individuals identifies 11 new susceptibility loci for Alzheimer’s disease. Nat Genet.

[34] Greenbaum L, Ravona-Springer R, Lubitz I, Schmeidler J, Cooper I, Sano M, Silverman JM, Heymann A, Beeri MS (2016). Potential contribution of the Alzheimer's disease risk locus BIN1 to episodic memory performance in cognitively normal type 2 diabetes elderly. Eur Neuropsychopharmacol.

[35] Halle A, Hornung V, Petzold GC, Stewart CR, Monks BG, Reinheckel T, Fitzgerald KA, Latz E, Moore KJ, Golenbock DT (2008). The NALP3 inflammasome is involved in the innate immune response to amyloid-beta. Nat Immunol.

[36] Heneka MT, Kummer MP, Stutz A, Delekate A, Schwartz S, Vieira-Saecker A, Griep A, Axt D, Remus A, Tzeng TC (2013). NLRP3 is activated in Alzheimer's disease and contributes to pathology in APP/PS1 mice. Nature.

[37] Butovsky O, Jedrychowski MP, Moore CS, Cialic R, Lanser AJ, Gabriely G, Koeglsperger T, Dake B, Wu PM, Doykan CE (2014). Identification of a unique TGF-beta-dependent molecular and functional signature in microglia. Nat Neurosci.

[38] Hikami K, Kawasaki A, Ito I, Koga M, Ito S, Hayashi T, Matsumoto I, Tsutsumi A, Kusaoi M, Takasaki Y (2011). Association of a functional polymorphism in the 3′-untranslated region of SPI1 with systemic lupus erythematosus. Arthritis Rheum.

[39] Speliotes EK, Willer CJ, Berndt SI, Monda KL, Thorleifsson G, Jackson AU, Lango Allen H, Lindgren CM, Luan J, Magi R (2010). Association analyses of 249,796 individuals reveal 18 new loci associated with body mass index. Nat Genet.

[40] Strawbridge RJ, Dupuis J, Prokopenko I, Barker A, Ahlqvist E, Rybin D, Petrie JR, Travers ME, Bouatia-Naji N, Dimas AS (2011). Genome-wide association identifies nine common variants associated with fasting proinsulin levels and provides new insights into the pathophysiology of type 2 diabetes. Diabetes.

[41] Hernandez GD, Solinsky CM, Mack WJ, Kono N, Rodgers KE, Wu CY, Mollo AR, Lopez CM, Pawluczyk S, Bauer G (2020). Safety, tolerability, and pharmacokinetics of allopregnanolone as a regenerative therapeutic for Alzheimer's disease: a single and multiple ascending dose phase 1b/2a clinical trial. Alzheimers Dement (N Y).

[42] Luchetti S, Huitinga I, Swaab DF (2011). Neurosteroid and GABA-A receptor alterations in Alzheimer’s disease, Parkinson’s disease and multiple sclerosis. Neuroscience.

[43] Castillo AF, Orlando U, Helfenberger KE, Poderoso C, Podesta EJ (2015). The role of mitochondrial fusion and StAR phosphorylation in the regulation of StAR activity and steroidogenesis. Mol Cell Endocrinol.

[44] Chang IY, Kim JK, Lee SM, Kim JN, Soh J, Kim JW, Yoon SP (2009). The changed immunoreactivity of StarD6 after pilocarpine-induced epilepsy. Neuroreport.

[45] Yin J, Feng W, Yuan H, Yuan J, Wu Y, Liu X, Jin C, Cheng Z (2019). Association analysis of polymorphisms in STARD6 and near ECHDC3 in Alzheimer's disease patients carrying the APOE epsilon4 allele. Neuropsychiatr Dis Treat.

[46] Kanekiyo T, Xu H, Bu G (2014). ApoE and Abeta in Alzheimer's disease: accidental encounters or partners?. Neuron.

[47] Long JM, Holtzman DM (2019). Alzheimer disease: an update on pathobiology and treatment strategies. Cell.

[48] Yin Y, Wang Z (2018). ApoE and neurodegenerative diseases in aging. Adv Exp Med Biol.

[49] Juan C, Yan X, Chenglin G, Runxuan W, Zulun L. Genes polymorphism of BIN1 and ApoE in patients with amnestic mild cognitive impairment from Enshi Tujia area. Natl Med J China. 2018;98(17):1322–6. 10.3760/cma.j.issn.0376-2491.2018.17.008 29764032

[50] Rosenbaum PR, Rubin DB (1983). The central role of the propensity score in observational studies for causal effects. Biometrilc.

