# Supplementary material for: Evaluation of mild cognitive impairment genetic susceptibility risks in a Chinese population
Source: BMC Psychiatry. 2022 Feb 8;22:93. doi: 10.1186/s12888-022-03756-y (PMC8822756; doi:10.1186/s12888-022-03756-y)
Supplement: Supplementary file 1 — Additional file 1: Supplementary Table 5. Detailed information of SNPs in fourteen GWAS-linked genesa. [file 12888_2022_3756_MOESM1_ESM.docx]

Table 5 Detailed information of SNPs in fourteen GWAS-linked genes^a^

| Gene | SNPs | Allele | Chr. | Position | Annotation |
| --- | --- | --- | --- | --- | --- |
| *BIN1* | rs6733839 | C/T | 2 | 127135234 | regulatory region variant |
|  | rs7561528 | G/A | 2 | 127132061 | intergenic variant |
| *RIN3* | rs10498633 | C/T | 14 | 92460608 | intron variant |
| *PICALM* | rs10792832 | G/A | 11 | 86156833 | downstream gene variant |
| *SPI1* | rs1057233 | T/C | 11 | 47354897 | 3 prime UTR variant |
| *TMP21* | rs12435391 | G/A | 14 | 75135034 | intron variant |
| *MTHFR* | rs1801133 | C/T | 1 | 11796321 | missense variant |
| *TMEM106B* | rs1990622 | C/T | 7 | 12244161 | downstream gene variant |
| *MC1R* | rs2228479 | G/A | 16 | 89919532 | missense variant |
| *CENPO* | rs6669072 | C/T | 1 | 90781788 | non coding transcript exon variant |
| *PVRL2* | rs6859 | G/A | 19 | 44878777 | 3 prime UTR variant |
| *STARD6* | rs10164112 | C/T | 18 | 54355010 | intron variant |
| *APOE* | rs7920721 | A/G | 10 | 11678309 | upstream gene variant |
|  | rs429358 | T/C | 19 | 44908684 | missense variant |
|  | rs7412 | C/T | 19 | 44908822 | missense variant |
| *KL* | rs9536314 | T/C | 13 | 33054001 | missense variant |
| *BZRAP1-AS1* | rs2632516 | G/C | 17 | 58331728 | non coding transcript exon variant |
| *PFDN1/*  *HBEGF* | rs11168036 | G/T | 5 | 140327854 | downstream gene variant |

a: data set was obtained from 1000 Genomes database (<http://asia.ensembl.org/>)
