# Supplementary material for: Evaluation of mild cognitive impairment genetic susceptibility risks in a Chinese population
Source: BMC Psychiatry. 2022 Feb 8;22:93. doi: 10.1186/s12888-022-03756-y (PMC8822756; doi:10.1186/s12888-022-03756-y)
Supplement: Supplementary file 2 — Additional file 2: Fig. 1 OR (95%CI) forest map of the effect of gene polymorphism on MCI. [file 12888_2022_3756_MOESM2_ESM.docx]

Fig.1 OR (95%CI) forest map of the effect of gene polymorphism on MCI
